# GenomeQC: a quality assessment tool for genome assemblies and gene structure annotations

**DOI:** 10.1186/s12864-020-6568-2

**Published:** 2020-03-02

**Authors:** Nancy Manchanda, John L. Portwood, Margaret R. Woodhouse, Arun S. Seetharam, Carolyn J. Lawrence-Dill, Carson M. Andorf, Matthew B. Hufford

**Affiliations:** 1 Department of Ecology, Evolution and Organismal Biology, Iowa State University, Ames, IA 50011 USA; 2 USDA-ARS Corn Insects and Crop Genetics Research Unit, Ames, IA 50011 USA; 3 Genome Informatics Facility, Iowa State University, Ames, IA 50011 USA; 4 Department of Genetics, Development and Cell Biology, Iowa State University, Ames, IA 50011 USA; 5 Department of Agronomy, Iowa State University, Ames, IA 50011 USA

**Keywords:** R, Shiny, Genome assembly, Gene annotations, Web interface, Docker containers

## Abstract

**Background:**

Genome assemblies are foundational for understanding the biology of a species. They provide a physical framework for mapping additional sequences, thereby enabling characterization of, for example, genomic diversity and differences in gene expression across individuals and tissue types. Quality metrics for genome assemblies gauge both the completeness and contiguity of an assembly and help provide confidence in downstream biological insights. To compare quality across multiple assemblies, a set of common metrics are typically calculated and then compared to one or more gold standard reference genomes. While several tools exist for calculating individual metrics, applications providing comprehensive evaluations of multiple assembly features are, perhaps surprisingly, lacking. Here, we describe a new toolkit that integrates multiple metrics to characterize both assembly and gene annotation quality in a way that enables comparison across multiple assemblies and assembly types.

**Results:**

Our application, named GenomeQC, is an easy-to-use and interactive web framework that integrates various quantitative measures to characterize genome assemblies and annotations. GenomeQC provides researchers with a comprehensive summary of these statistics and allows for benchmarking against gold standard reference assemblies.

**Conclusions:**

The GenomeQC web application is implemented in R/Shiny version 1.5.9 and Python 3.6 and is freely available at https://genomeqc.maizegdb.org/ under the GPL license. All source code and a containerized version of the GenomeQC pipeline is available in the GitHub repository https://github.com/HuffordLab/GenomeQC.

## Background

Over the past few decades, numerous plant genome assemblies have been generated, ranging in size from 63 Mb in *Genlisea aurea* [1] to 22 Gb in *Pinus taeda* [2]. The genomic resources generated from such projects have contributed to the development of improved crop varieties, enhanced our understanding of genome size, architecture, and complexity, and uncovered mechanisms underlying plant growth and development [3, 4]. With the declining cost of sequence, the number of genome assemblies has increased exponentially (Additional file 1: Figure S1). The NCBI assembly database [5] currently hosts more than 800 plant genome assemblies with varying degrees of contiguity and increasingly includes multiple genome assemblies per species (Additional file 1: Figure S2).

The growing number of assemblies and gene annotations has necessitated the development of metrics that can be used to compare their quality. Such metrics also allow evaluation of the performance of various assembly and annotation methods using the same data. Length metrics (N50/NG50 and L50/LG50 values) provide a standard measure of assembly contiguity [6]. The most commonly reported N50/NG50 values are calculated for the 50% threshold, but NG(X) plots across all thresholds (1–100%) provide a more complete picture [6]. Annotation quality metrics include number of gene models, exons per gene model, and the average lengths of genes, exons and transcripts [7]. Such length and count metrics are useful, but they do not fully capture the completeness of assemblies.

Completeness is better gauged using a set of genes that are universally distributed as orthologs across particular clades of species [8]. A summary of complete single-copy, duplicated, fragmented, and missing Benchmarking Universal Single-Copy Orthologs (BUSCO) genes is often provided as a quantitative measure of genome completeness based on expected gene content. While BUSCO is limited to assessment of the gene space, the LTR Assembly Index (LAI [9];) is capable of gauging completeness in more repetitive genomic regions by estimating the percentage of intact LTR retroelements. LAI is particularly useful for assessing plant genome assemblies, which are often largely comprised of repeats. Recently, dramatic increases in the completeness of repetitive portions of plant genomes have been achieved due to improvements in long-read data [9].

Here, we describe an easy-to-use and interactive web framework based on the R/Shiny package [10] that integrates a suite of quantitative measures to characterize genome assemblies and annotations. Our application, named GenomeQC, provides researchers with a summary of these statistics and allows for benchmarking against gold standard reference assemblies. We have also developed a Docker container of the GenomeQC pipeline that calculates these metrics and supports analysis of large (> 2.5Gb) genomes.

## Implementation

### Comparison with similar software programs

Although several tools exist for evaluating and visualizing the quality of genome assemblies, they are often challenging to install and configure, do not support assessment of gene structure annotations, and do not determine the completeness of the repetitive fraction of the genome based on LTR retrotransposon content. We tested the GenomeQC tool along with two other genome assembly evaluation tools, QUAST-LG [11] and REAPR [12] on three maize genome datasets (B73_v4, Mo17 and W22) as input. Table 1 shows the comparison of the output metrics generated by each tool along with their run time on the test datasets. The full details of the outputs and the datasets used for benchmarking the tools are included in the Additional file 2 (Table S1, Table S2, Table S3, Table S4, Table S5, Table S6) along with parameters and commands used in running these tools.

**Table 1:** Comparison of the key metrics and features of the GenomeQC tool with two other assembly evaluation tools QUAST-LG and REAPR

Metrics	GenomeQC	QUAST-LG	REAPR
Reference-free standard metrics (with just the genome assembly as input)	Yes	Yes	Yes
Metric based on gene space completeness (BUSCO)	Yes	Yes	No
BUSCO datasets and training options	BUSCO profile datasets: 34, Augustus species: all	BUSCO profile datasets: 3 (Fungal, Eukaryote, bacterial), Augustus species: 1 (fly)	No
Metrics based on whole genome alignment to reference genome assembly	No	Yes	No
Metrics based on mapping raw reads to the assembly	No	Yes	Yes
Metrics based on repeat space completeness (LAI)	Yes	No	No
Vector contamination check	Yes	No	No
Assessment of gene structure annotations set	Yes	No	No
Web server for the program	Yes	No	No
Dockerfile availability	Yes	No	No
Runtime (CPU hours)	~ 1116	~ 2340	~ 2556

## Design concept

### Workflow of the web application

**Fig. 1:**
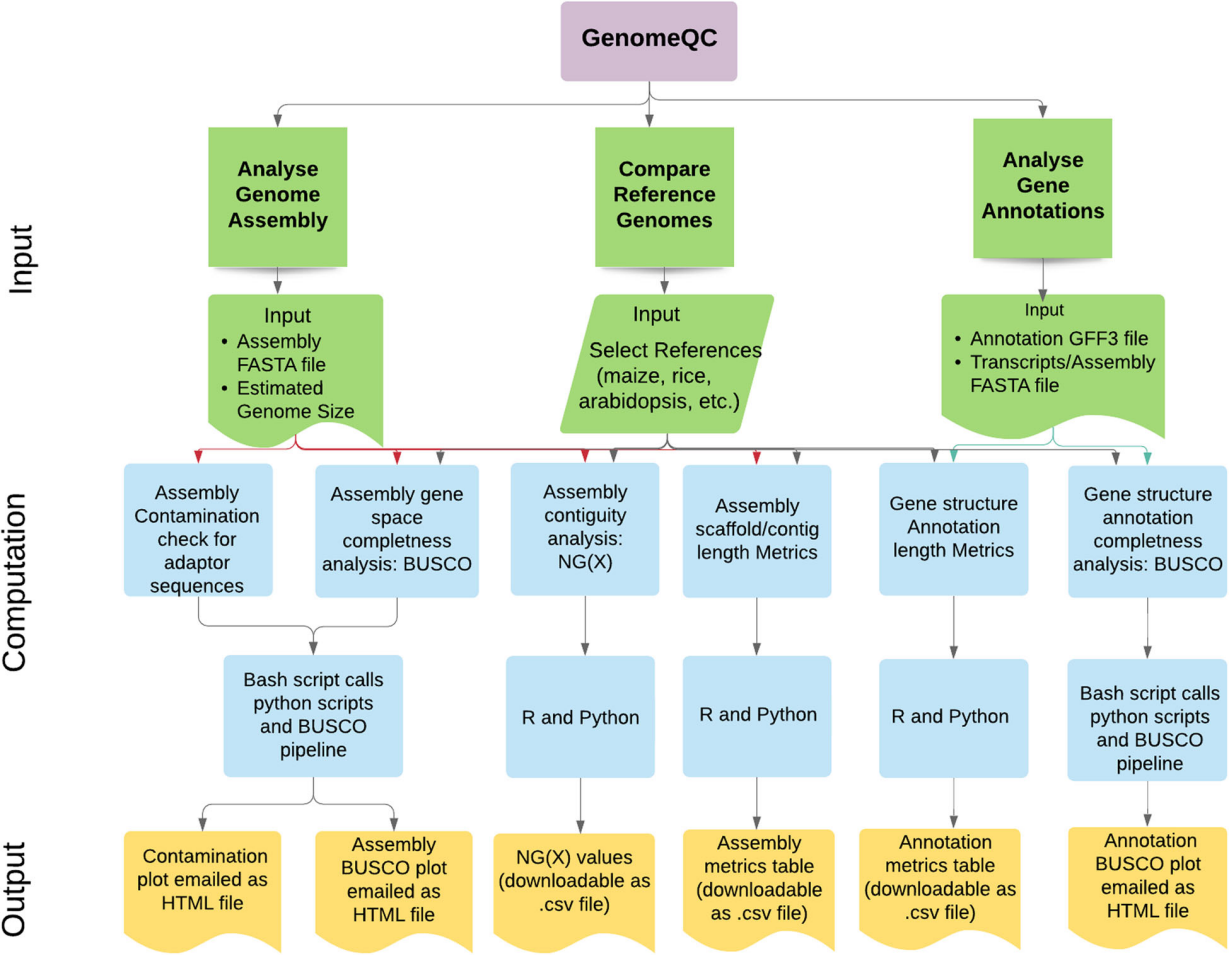
Workflow of the web application. The interface layer of the web application is partitioned into 3 sections: comparing reference genomes, analyzing genome assembly and analyzing gene structure annotations (green). Each of these sections has an input widget panel for file uploads and parameter selection (green). The input parameters and the uploaded data files are then analyzed for contiguity, gene space and repeat space completeness, and contamination check (blue) using bash, R and python scripts (blue) and the different metrics and plots are displayed through the output tabs (yellow)

The web-application of the GenomeQC tool (Fig. 1) has three sections:

#### Analyze Genome Assembly

##### Input layer

This allows the user to upload a maximum of two genome assemblies for analysis. Users also have the option to benchmark the quality of the uploaded genome assembly with the gold reference genomes by selecting the names from the drop-down list. Other inputs that the user needs to provide for this section include: the label for the genome assembly plots, estimated genome size, datasets and species name for BUSCO analysis and email address to which the plots will be sent.

##### Computation layer

This calculates standard length and number metrics like N50, L50, vector contamination check and gene set completeness.

To calculate the standard length metrics N50, L50, NG(X) values for the user uploaded assembly, two custom python scripts NG.py and assembly_stats.py are employed. The gene space completeness analysis of the genome assembly is performed using the BUSCO package version 3.0.2 [8] with genome mode. For vector contamination check, custom script contamination.py is used which implements a python wrapper for the NCBI BLAST+ program [13] blastn and a modified version of the taxify script from the blobtools package v1.1 [14] to blast (with parameters: task = “megablast”, max_target_seqs = 1, max_hsps = 1, evalue = 1e-25) the input contigs/scaffold sequences in the uploaded genome assembly against the UniVec Database [15] and add the taxon ids to the blast hits. All the plots are generated using the R package ggplot2 and python modules pandas and plotly.

##### Output layer

The output layer of the interface displays the NG(X) plot and the interactive assembly metrics table. The BUSCO and contamination plots are emailed to the user at the provided email address.

#### Compare Reference Genomes

##### Input layer

The input widget of this section takes two parameters: name of one or more reference genome assemblies and the user’s email address.

##### Computation layer

The reference genome metrics are pre-calculated using the same custom scripts NG.py and assembly_stats.py and the BUSCO package version 3.0.2. The R package ggplot2 and a custom python script (modules pandas and plotly) are used to plot the pre-computed reference metrics.

The parameters used for the computation of metrics for the reference genomes of the different plant species are provided in the GenomeQC user guide accessible at the GitHub repository.

##### Output layer

The output layer of the interface displays the NG(X) plot and the interactive assembly metrics table. The BUSCO assembly and annotation plots are emailed to the user at the provided email address.

#### Analyze Genome Annotation

##### Input layer

This allows the user to upload a gene structure annotation set, genome assembly and transcript file (optional) for analysis. Users also have the option to benchmark the quality of the uploaded gene annotations with the gold reference genomes by selecting the names from the drop-down list. Other inputs that the user needs to provide for this analysis section include: labels for the plots and table, dataset names for BUSCO analysis and email address to which the plots will be sent.

##### Computation layer

Once the required files and parameters are provided to the tool, it computes the length and count metrics for different features of the GFF file using the custom python script gff_stats.py and assesses the completeness of the gene set based on a conserved set of orthologs using the BUSCO package version 3.0.2 with transcriptome mode.

##### Output layer

The output layer displays the interactive annotation metrics table file. The BUSCO stack plots are emailed to the user at the provided email address.

##### Workflow of the docker application

**Fig. 2:**
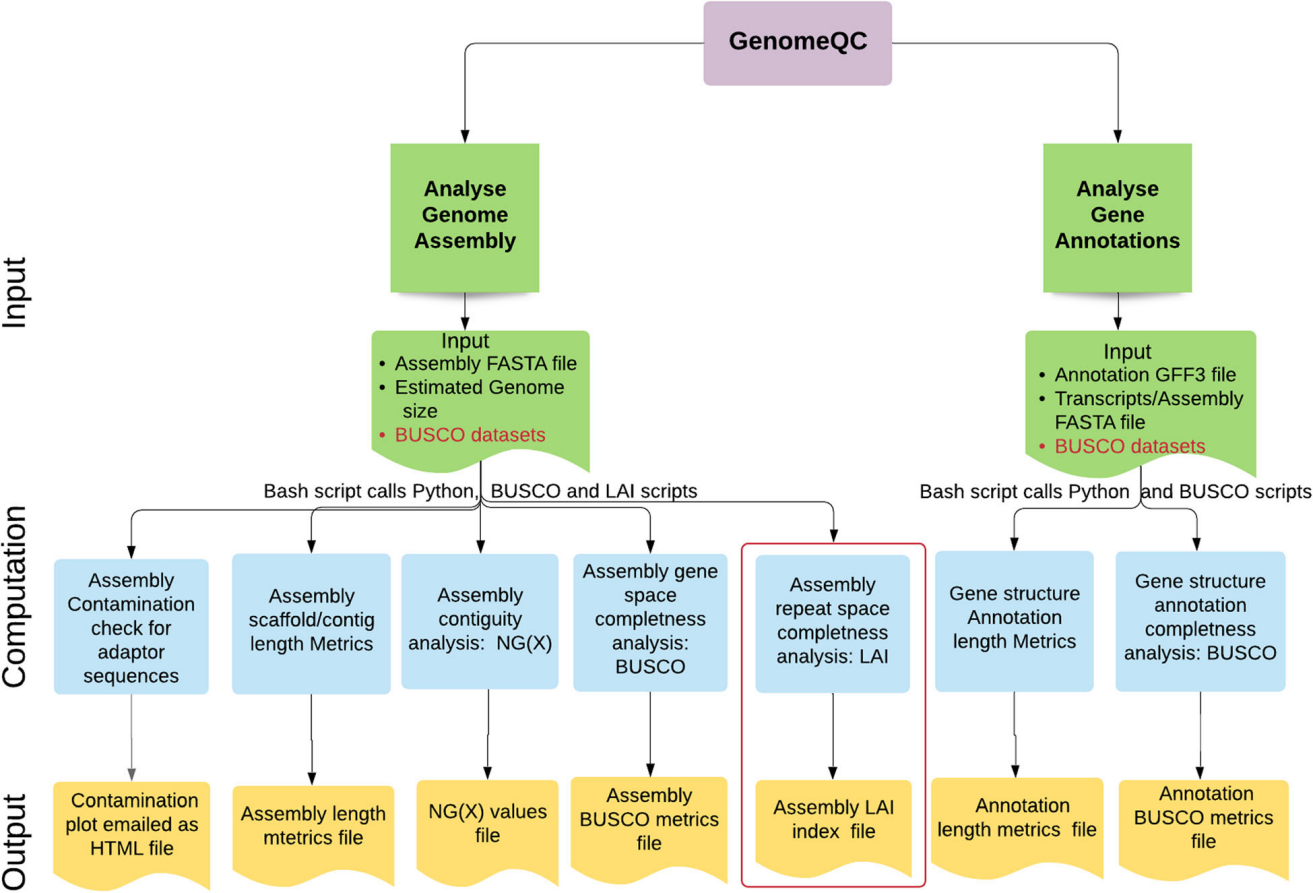
Workflow of the docker image of the GenomeQC pipeline. The containerized version of the GenomeQC pipeline requires BUSCO datasets (highlighted in red) as input in addition to the other input parameters and files (green) required by the web application. Additionally, the containerized version allows computation of the LAI index for the input genome assembly (highlighted in the red box)

There are two docker files: one for analyzing the genome assembly and a second for analyzing the genome annotation file (Fig. 2).

#### Analyze Genome Assembly

##### Input

The docker pipeline takes as input: genome assembly in FASTA format, estimated genome size in Mb, BUSCO datasets and species name, email address and the name of the output files and directory.

##### Computation

The pipeline computes the various relevant assessment metrics like N50, L50, NG(X) values, BUSCO gene space completeness metrics and vector contamination check. In addition to these metrics, the docker pipeline provides the functionality to compute LTR Assembly Index (LAI) of the input genome assembly to assess the repeat space completeness of the assembled genome sequence. To calculate the standard length and count metrics N50, L50 etc. and the NG(X) values for the user input assembly, two custom python scripts NG.py and assembly_stats.py are employed. The gene space completeness analysis of the genome assembly is performed using the BUSCO package version 3.0.2 with genome mode. For vector contamination check, custom script contamination.py is used that implements the python wrapper for the NCBI BLAST+ program blastn (with parameters: task = “megablast”, max_target_seqs = 1, max_hsps = 1, evalue = 1e-25) to blast the input contigs/scaffold sequences in the uploaded genome assembly against the UniVec Database and a modified version of the taxify script (from the blobtools package v1.1) to add the taxon ids to the blast hits. To calculate the LAI score for the input genome assembly, the pipeline uses the software package LTR retriever v2.8.2 [16]. This program is designed to identify intact LTR retrotransposons with high accuracy and sensitivity. This set of high confidence LTR retrotransposons are then used to assess the repeat space completeness of the assembly by calculating the percentage of fully-assembled LTR retrotransposons in the assembled genome sequence.

##### Output

The pipeline generates the following output files and directories:

1. Output file (text file format) containing the NG(X) values which could be easily plotted in R or Excel to generate the NG(X) graph.
2. Assembly metrics output file (text file format) contains all the standard metrics like N50, L50, total number of bases, %N, etc.
3. Vector contamination plot in HTML format and the associated blast hits.
4. BUSCO output directory containing the summary text file for the number of complete, fragmented and missing BUSCO genes identified in the input genome assembly.
5. LAI output file (.out. LAI) containing the LAI score for the input assembly.

#### Analyze Genome Annotation

##### Input

The docker pipeline takes as input: genome annotation file in GFF format, and transcripts file in FASTA format BUSCO datasets, and the name of the output files and directory.

##### Computation

The pipeline computes the various relevant assessment metrics as computed by the web-server including number and length of gene models, exons, etc. and the BUSCO gene space completeness metrics. Custom python script gff_stats.py is employed to calculate the different gene model statistics for the input annotation GFF file. The gene space completeness analysis of the input genome annotations is performed using the BUSCO package version 3.0.2 with transcriptome mode.

##### Output

The pipeline generates the following output files and directories:

1. Annotation metrics output file (text file format) that contains the relevant statistics on the different features of the GFF file like number of gene models, exons, transcripts etc.
2. BUSCO output directory containing the summary text file for the number of complete, fragmented and missing BUSCO genes identified in the input genome annotation set.

All the packages used in the web-application and docker pipeline are mentioned in Table 2, Table 3 and Table 4.

**Table 2:** R packages used in the GenomeQC web-application

R package	Short Description
Shiny version 1.5.9	Package to build interactive web applications with R
Tools [17]	Package for file utilities
Seqinr [18]	Package for handling biological sequence data
Biostrings [19]	Package for manipulating biological sequences
R.utils [20]	Package for handling gunzipped files
Tidyverse [21]	Package for formatting and plotting data
Gridextra [22], grid [22], cowplot [23]	Package provides graphical layout capabilities to R
Reshape [24]	Package for formatting and aggregating the data
shinyWidgets [25]	Package for customizing input widgets in R shiny applications
shinyBS [26]	Package for adding action and toggle buttons and popover to input or output
Promise, future and multisession [27]	Package that provides async programming in R to handle long-running operations that run in the background

**Table 3:** Python packages used in the GenomeQC web-application and standalone application

Python package	Short Description
Sys, os, argparse, re, traceback, subprocess, collections [28]	Standard libraries and modules that are distributed with the python installation. These packages provide access to system-specific parameters and functions, functionality to interact with the operating systems, parse command line arguments, etc.
Bio [29]	Provides functionality for computation of biological sequence data
Statistics [30]	Provides functionality for mathematical computation
Numpy [31]	Fundamental package for scientific computing
Bio.Blast.Applications [13]	Provides the NCBI BLAST command line utility for python
Iglob [32]	Package to find files in the directory through pattern matching
Pandas [33]	Python library for data analysis and manipulation
Plotly.offline plotly.graph_objs [34]	Python package for creating interactive plots
Matplotlib [35]	Provides plotting functionality to python for data visualization
email.mime.text, email.mime.application email.mime.multipart, smtplib [36]	Python package that provides email handling functionality to python

**Table 4:** External tools used in the GenomeQC web-application and standalone application. Note that the LTR retriever package is included in the standalone application only

External tools	Short Description
BUSCO v3.0.2 Dependencies: NCBI BLAST+ v2.28.0 Augustus v3.2.1 [37] HMMER v3.1b2 [38]	BUSCO Package is used for assessing gene space completeness using an ortholog set of conserved genes. BUSCO assessment of genome assembly involves constructing gene models from the candidate regions identified by tblastn searches against the consensus sequences. BUSCO pipeline uses AUGUSTUS de novo gene predictor to construct the gene models. These gene predictions are then used by HMMER which classifies the matches of gene predictions with the BUSCO lineage profiles as complete and single copy (C&S), duplicated (D), fragmented (F) or missing (M).
Gffread 0.9.12 [39]	Gffread is a Cufflinks utility that is used to extract the transcript sequences given the genome fasta file and annotation GFF file. (http://ccb.jhu.edu/software/stringtie/gff.shtml)
NCBI UniVec Database	Database of vector sequences, adaptors, linkers and primer sequences used in DNA cloning
Taxify module, BtIO.py, BtLog.py (Blobtools v1.1)	This script is used to add NCBI TaxID to the blast hits of the input contig/scaffold sequences to the UniVec Database
LTR retriever v2.8.2 Dependencies: NCBI BLAST+ 2.9.0 RepeatMasker 4.0.9 [40] HMMER 3.2.1 CDHIT 4.8.1 [41] LTRFINDER parallel [42] LTRharvest 1.5.10 [43]	LTR retriever package is used to calculate LTR Assembly index (LAI)^23^ of the input genome assembly. LTRharvest and LTRFinder tools are first used to obtain retrotransposon candidates. LTR retriever package filters out false positives and generates high confidence intact LTR retrotransposons from the candidate sequences. Repeat Masker is used for whole genome LTR annotation to annotate all possible LTR-RTs present in the genome. LAI is finally calculated as the percentage of the total length of intact LTR retrotransposons present in the assembled genome sequence.

## Results

### Input files

Two files are required as input for GenomeQC analysis.

“Genome Assembly File” is a sequence file in the standard FASTA format. The file should be gunzipped compressed (.gz) before uploading it to the web-application. The maximum upload limit for the assembly file is 1Gb.

“Genome Structure Annotation File” is a tab separated text file in GFF/GTF format [17]. The file should be gunzipped compressed (.gz) before uploading it to the web-application.

#### Optional file

“Transcript FASTA file”: BUSCO analysis of structural annotations requires a transcript file in FASTA format as input. Thus, the user could either directly upload a transcript (DNA nucleotide sequences) file in compressed (.gz) FASTA format or the tool could extract the transcript sequences from the uploaded assembly and annotation files using the gffread utility v0.9.12 [18]. Currently the tool is configured to first use the information from a transcripts file if provided by the user. If the user does not upload the transcripts file, the tool will check whether the sequence IDs in the first column of the GFF file correspond to the headers in the FASTA file. If there is a discrepancy, the tool will print an error message. Otherwise, the BUSCO job will be submitted.

### Interface design

The tool’s analysis interface is organized into three sections for three types of analysis.

The “Compare reference genomes” section outputs various pre-computed assembly and annotation metrics from a user-selected list of reference genomes.

The “Analyze your genome assembly” section provides the user the option to perform analysis on their genome assembly as well as benchmark the quality of their genome assembly using pre-computed metrics from gold standard reference genomes.

The “Analyze your genome annotation” section provides the user the option to perform analysis on their genome annotations as well as benchmark their analysis versus pre-computed reference genomes.

### Output tabs

The “Assembly NG(X) Plot” tab calculates NG values for an uploaded assembly based on the input estimated genome size at different integer thresholds (1–100%) and generates a plot showing the thresholds on the x-axis and the corresponding log-scaled scaffold or contig lengths on the y-axis. Genome assemblies with larger scaffold/contig lengths across NG(X) thresholds are more contiguous.

The NG(X) values can be downloaded as a .csv file and the plot can be saved in png format by right clicking on the plot.

The “Assembly Metrics Table” and the “Annotation Metrics Table” tabs calculate various length and count metrics for the uploaded assembly and annotation files and outputs interactive tables with pop-up plots based on row selection. These tabs provide the user with quick summaries of standard assembly and annotation metrics. These tables can be downloaded as comma separated files.

The “Assembly BUSCO and Contamination Plots” tab: calculates and emails BUSCO scores for the uploaded genome assembly and compares it with the pre-computed values of the user-selected reference genomes. A high quality genome assembly is expected to contain a higher number of complete and single copy BUSCO genes (C&S) and a lower number of missing (M) or fragmented (F) BUSCO genes [8]. These plots are emailed as png and html files. The HTML file can be opened in a chart studio and customized.

**Fig. 3:**
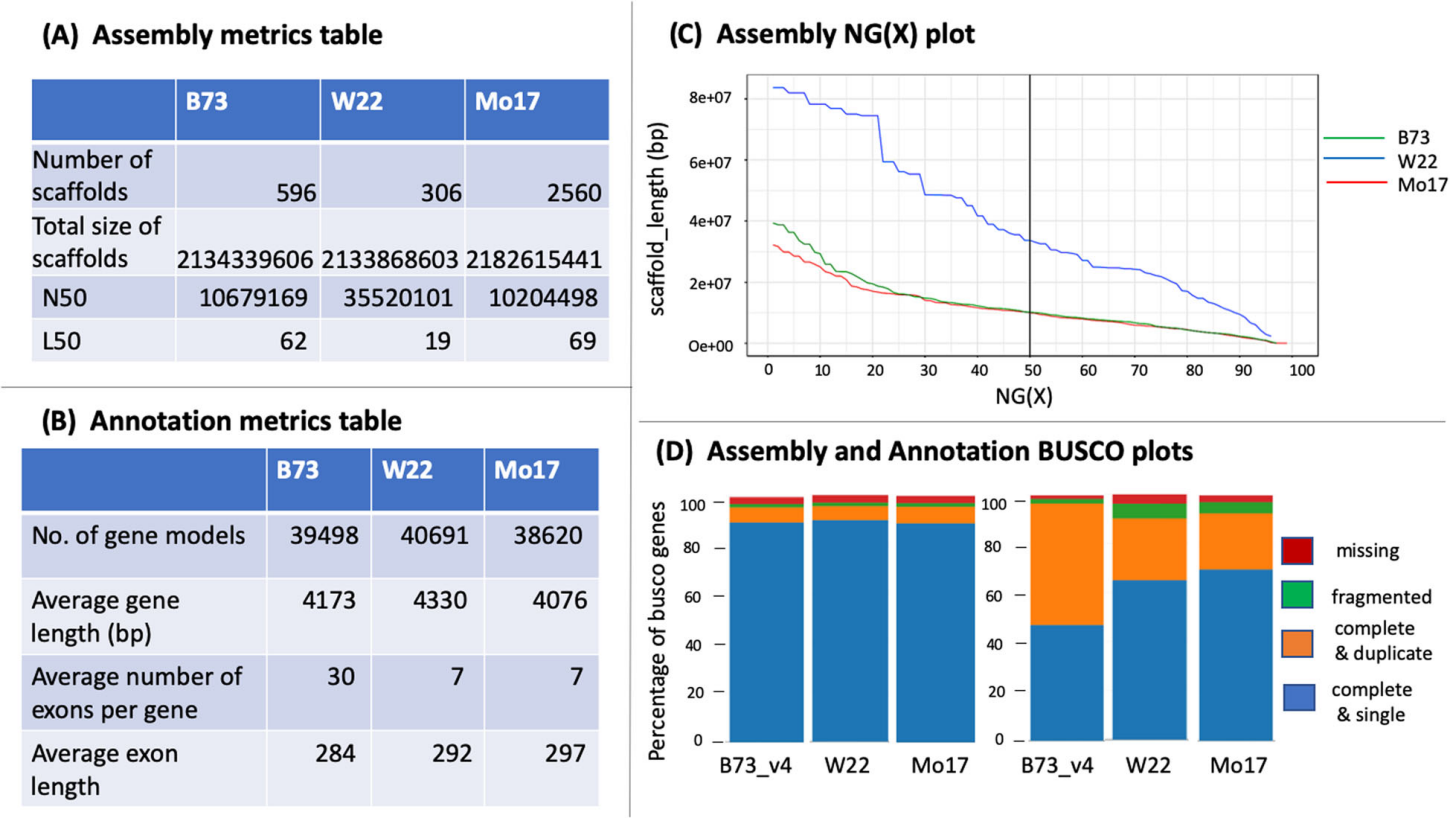
Summaries and graphical output by GenomeQC. **a** and **b** include standard assembly and annotation metrics generated for maize reference lines B73, W22 and Mo17. **c** is an NG(X) graph in which the x-axis charts NG(X) threshold values (1 to 100%) and the y-axis shows scaffold lengths. Each curve represents scaffold lengths of assemblies at different NG levels with a bold vertical line at the commonly used NG50 value. **d** shows the relative proportion of complete and single copy (blue), complete and duplicated (orange), fragmented (green), and missing (red) Benchmark Universal Single Copy Ortholog (BUSCO) genes identified for the assembly (left) and gene annotation set (right) of the above-mentioned maize lines

The “Annotation BUSCO plot” tab calculates and emails the BUSCO scores for the uploaded genome annotations and compares it with pre-computed values of the user-selected reference genomes. BUSCO and contamination plots are also emailed as html files. Figure 3 shows the summaries and graphical outputs generated by the GenomeQC web application.

## Conclusions

GenomeQC provides a user-friendly web framework for calculating contiguity and completeness metrics for genome assemblies and annotations. The web application is optimized to compute metrics for small to medium-sized genomes with an upper limit of 2.5 Gb (the approximate size of the maize genome). However, the containerized version of the pipeline available through our GitHub repository can be used to calculate metrics for larger genomes. In addition to standard length metrics such as N50 and L50, GenomeQC assesses gene- and repeat-space completeness of an input genome assembly and screens for both vector and adapter contamination, a standard check implemented by NCBI before accepting new assemblies into the database. The web application of GenomeQC calculates standard metrics on the fly with just the genome assembly as input, requiring no additional computational resources or software installation. Optional annotation assessments are also performed by GenomeQC when gene predictions are provided as input (in GFF format). The report includes standard statistics of the gene model features like the number and size of gene models, exons, transcripts, etc. and performs quality assessment of the gene structure annotations using the BUSCO ortholog gene set. Finally, GenomeQC allows researchers to benchmark their metrics relative to gold standard reference genomes. These utilities should prove useful as the practice of genome assembly increasingly becomes a routine component of a biologists’ toolbelt.

## Availability of source code and requirements

Project name: GenomeQC.

Project home page:

https://github.com/HuffordLab/GenomeQC

Operating system(s): platform independent

Programming language: R, R shiny, Python, Shell script

Other requirements: Docker engine

License: Any restrictions to use by non-academics: None

## Supplementary information

**Additional file 1: Figure S1.** Exponential growth in the number of plant genome assemblies deposited in the NCBI Assembly database from November 2004 through December 2018. **Figure S2.** Number of plant genome assemblies in the NCBI Assembly Database at each level of assembly contiguity.

**Additional file 2. **Information on the input data, parameters and output from the three tools: QUAST-LG, REAPR and GenomeQC. **Table S1.** QUAST-LG output with just the genome fasta file as input. **Table S2.** QUAST-LG output with reference genome as input**. Table S3.** QUAST-LG output with reads as input**. Table S4.** REAPR output**. Table S5.** GenomeQC assembly output. **Table S6.** GenomeQC annotation output.

## Data Availability

Not applicable.

## Abbreviations

BUSCO: Benchmark Universal Single Copy Orthologs
LAI: LTR Assembly Index
LTR: Long Terminal Repeats
