# Supplementary material for: GenomeQC: a quality assessment tool for genome assemblies and gene structure annotations
Source: BMC Genomics. 2020 Mar 2;21:193. doi: 10.1186/s12864-020-6568-2 (PMC7053122; doi:10.1186/s12864-020-6568-2)
Supplement: Supplementary file 1 — Additional file 1: Figure S1. Exponential growth in the number of plant genome assemblies deposited in the NCBI Assembly database from November 2004 through December 2018. Figure S2. Number of plant genome assemblies in the NCBI Assembly Database at each level of assembly contiguity. [file 12864_2020_6568_MOESM1_ESM.docx]

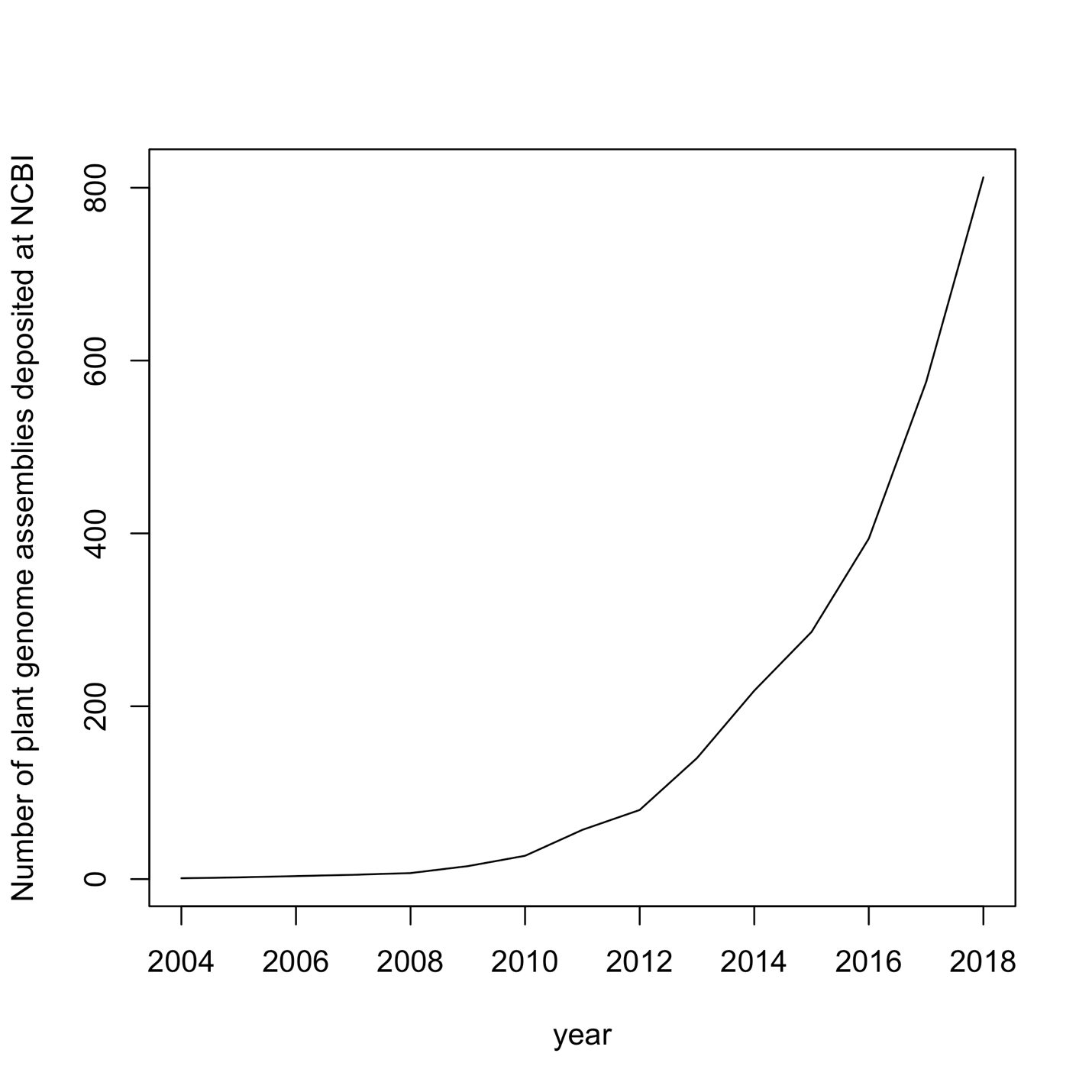


**Figure S1:** Exponential growth in the number of plant genome assemblies deposited in the NCBI Assembly database from November 2004 through December 2018.


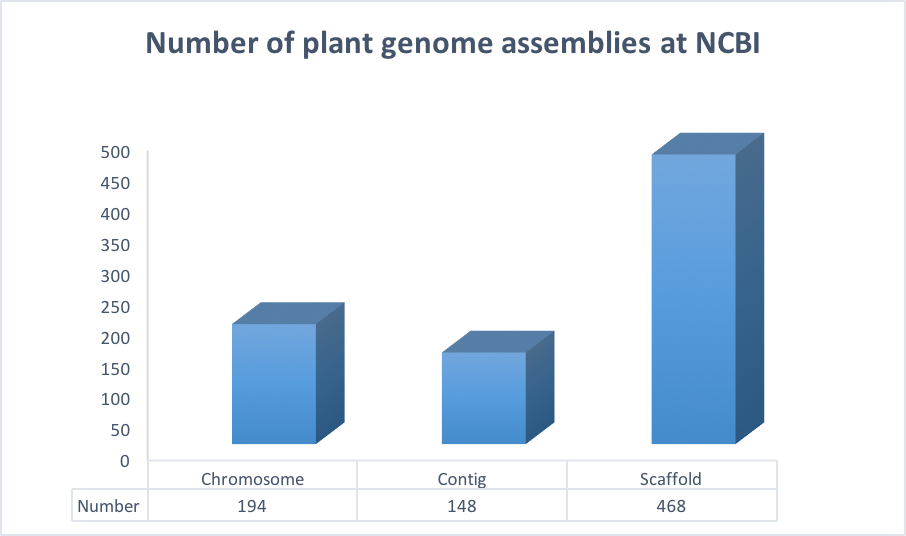


**Figure S2.** Number of plant genome assemblies in the NCBI Assembly Database at each level of assembly contiguity.
