# Supplementary material for: GenomeQC: a quality assessment tool for genome assemblies and gene structure annotations
Source: BMC Genomics. 2020 Mar 2;21:193. doi: 10.1186/s12864-020-6568-2 (PMC7053122; doi:10.1186/s12864-020-6568-2)
Supplement: Supplementary file 2 — Additional file 2. Information on the input data, parameters and output from the three tools: QUAST-LG, REAPR and GenomeQC. Table S1. QUAST-LG output with just the genome fasta file as input. Table S2. QUAST-LG output with reference genome as input. Table S3. QUAST-LG output with reads as input. Table S4. REAPR output. Table S5. GenomeQC assembly output. Table S6. GenomeQC annotation output. [file 12864_2020_6568_MOESM2_ESM.docx]

Comparison of existing tools section:

**Data used in the analysis:**

1. **Raw reads files:**

B73_v4: Illumina short reads were downloaded from NCBI (SRR2960981)

Mo17: Illumina short reads were downloaded from NCBI (SRR5826129, SRR5826130)

W22: We were not able to find short-read information data from NCBI for this genome assembly.

1. **Genome assembly files:**

**B73_v4:**

Fasta files (they were combined into a single file) used for Assembly metrics table and BUSCO score calculation.

Placed scaffolds: This file was created by splitting the AGP file based on the WGS coordinate information. (Source of AGP files: ftp://ftp.ncbi.nlm.nih.gov/genomes/genbank/plant/Zea_mays/all_assembly_versions/GCA_000005005.6_B73_RefGen_v4/GCA_000005005. 6_B73_RefGen_v4_assembly_structure/Primary_Assembly/assembled_chromosomes/AGP/).

Unplaced scaffolds (source: ftp://ftp.ncbi.nlm.nih.gov/genomes/genbank/plant/Zea_mays/all_assembly_versions/GCA_000005005.6_B73_RefGen_v4/GCA_000005005. 6_B73_RefGen_v4_assembly_structure/Primary_Assembly/unplaced_scaffolds/FASTA/)

Both placed and unplaced scaffolds were combined to generate the final scaffold file for B73_v4.

**Mo17:**

Fasta file used for Assembly metrics table and BUSCO score calculation.
Source of this file: provided by the Mo17 Sequencing Group (State Key Laboratory of Agrobiotechnology and National Maize Improvement Center, Department of Plant Genetics and Breeding, China Agricultural University, Beijing, China). This file could be downloaded from the CyVerse directory (https://de.cyverse.org/dl/d/E5EE609A-B0D1-4D6F-88A6-DA7D280C7FF8/Zm-Mo17-REFERENCE-CAU-1.0.fsa.gz).

**W22:**

Fasta files (they were combined into a single file) used for Assembly metrics table and BUSCO score calculation.
Placed scaffolds: This file was created by splitting the AGP file based on the WGS coordinate information. (Source of AGP files: [ftp://ftp.ncbi.nlm.nih.gov/genomes/genbank/plant/Zea_mays/all_assembly_versions/GCA_001644905.2_Zm-W22-REFERENCE-NRGENE- 2.0/GCA_001644905.2_Zm-W22-REFERENCE-NRGENE-2.0_assembly_structure/Primary_Assembly/assembled_chromosomes/AGP/](ftp://ftp.ncbi.nlm.nih.gov/genomes/genbank/plant/Zea_mays/all_assembly_versions/GCA_001644905.2_Zm-W22-REFERENCE-NRGENE-%202.0/GCA_001644905.2_Zm-W22-REFERENCE-NRGENE-2.0_assembly_structure/Primary_Assembly/assembled_chromosomes/AGP/)).

Unplaced scaffolds (source: ftp://ftp.ncbi.nlm.nih.gov/genomes/genbank/plant/Zea_mays/all_assembly_versions/GCA_001644905.2_Zm- W22-REFERENCE-NRGENE-2.0/GCA_001644905.2_Zm-W22-REFERENCE-NRGENE- 2.0_assembly_structure/Primary_Assembly/unplaced_scaffolds/FASTA/) were combined with the placed scaffolds extracted from the AGP file to generate the final scaffold file for W22_NRgene.

1. **Genome annotation files:**

**B73_v4:**

GFF file used for Annotation metrics table calculation.
Source of this file: <ftp://ftp.ensemblgenomes.org/pub/plants/release-36/gff3/zea_mays/>

**Transcript file (used for BUSCO analysis):**

The fasta file was used to extract transcript sequences using gffread.
Source of this file: ftp://ftp.ensemblgenomes.org/pub/plants/release-36/fasta/zea_mays/dna/

The transcript file is the output from the gffread program (used for extracting transcripts sequences from gff files).

./gffread -w B73_v4_transcripts.fasta -g Zea_mays.AGPv4.dna.toplevel.fa Zea_mays.AGPv4.36.gff3

**Mo17:**

GFF file used for Annotation metrics table calculation.
Source of this file: [https://download.maizegdb.org/Zm-Mo17-REFERENCE-CAU-1.0/](https://download.maizegdb.org/Zm-Mo17-REFERENCE-CAU-1.0/Zm-Mo17-REFERENCE-CAU-1.0_Zm00014a.1.gff3.gz)

**Transcript file (used for BUSCO analysis):**

The fasta file was used to extract transcript sequences using gffread. Source of this file: [https://download.maizegdb.org/Zm-Mo17-REFERENCE-CAU-1.0/](https://download.maizegdb.org/Zm-Mo17-REFERENCE-CAU-1.0/Zm-Mo17-REFERENCE-CAU-1.0.fa.gz)

The transcript file is used for BUSCO annotation score calculation.
./gffread -w Mo17_transcripts.fasta -g Zm-Mo17-REFERENCE-CAU-1.0.fsa Mo17.gff3

**W22:**
GFF file used for Annotation metrics table calculation.
Source of this file: [https://download.maizegdb.org/Zm-W22-REFERENCE-NRGENE-2.0/](https://download.maizegdb.org/Zm-W22-REFERENCE-NRGENE-2.0/Zm-W22-REFERENCE-NRGENE-2.0.scaffolds.fa.gz)

**Transcript file (used for BUSCO analysis):**
Source of this file: [https://download.maizegdb.org/Zm-W22-REFERENCE-NRGENE-2.0/](https://download.maizegdb.org/Zm-W22-REFERENCE-NRGENE-2.0/Zm-W22-REFERENCE-NRGENE-2.0_Zm00004b.1.gff3.gz)

**Commands used for the analysis:**

**QUAST-LG:**

Without reference genome (using just the genome fasta file as input):

B73_v4:

quast-lg.py --eukaryote --est-ref-size 2200000000 --debug --threads 36 --conserved-genes-finding --min-contig 0 --large B73_v4_scaffolds.fasta -o b73

Mo17:

quast-lg.py --eukaryote --est-ref-size 2200000000 --debug --threads 36 --conserved-genes-finding --min-contig 0 --large Zm-Mo17-Scaffolds-CAU-1.0.fa -o mo17

W22:

quast-lg.py --eukaryote --est-ref-size 2200000000 --debug --threads 36 --conserved-genes-finding --min-contig 0 –large W22_NRgene_scaffolds.fasta -o w22

**Table S1.** QUAST-LG output with just the genome fasta file as input

| **Metrics** | **B73_v4** | **Mo17** | **W22** |
| --- | --- | --- | --- |
| # contigs (>= 0 bp) | 596 | 2560 | 306 |
| # contigs (>= 1000 bp) | 596 | 2560 | 305 |
| # contigs (>= 5000 bp) | 596 | 2433 | 301 |
| # contigs (>= 10000 bp) | 591 | 2216 | 291 |
| # contigs (>= 25000 bp) | 588 | 1425 | 218 |
| # contigs (>= 50000bp) | 553 | 699 | 163 |
| Total length (>= 0 bp) | 2134339606 | 2182615441 | 2133868603 |
| Total length (>= 1000 bp) | 2134339606 | 2182615441 | 2133867892 |
| Total length (>= 5000 bp) | 2134339606 | 2182208430 | 2133862796 |
| Total length (>= 10000 bp) | 2134301844 | 2180557762 | 2133788195 |
| Total length (>= 25000 bp) | 2134248774 | 2166421525 | 2132523330 |
| Total length (>= 50000 bp) | 2132786002 | 2142273918 | 2130603434 |
| # contigs | 596 | 2506 | 301 |
| Largest contig | 39317442 | 32176138 | 83688764 |
| Total length | 2134339606 | 2182500096 | 2133862796 |
| GC (%) | 46.87 | 46.83 | 46.75 |
| N50 | 10679169 | 10204498 | 35520101 |
| NG50 | 10214929 | 9989738 | 33636442 |
| N75 | 6157473 | 5393738 | 22811713 |
| NG75 | 5423395 | 5254770 | 21892233 |
| L50 | 62 | 69 | 19 |
| LG50 | 66 | 70 | 20 |
| L75 | 129 | 144 | 39 |
| LG75 | 137 | 146 | 41 |
| # N’s per 100 kbp | 1438.7 | 1609.15 | 1903.29 |
| Complete & single copy BUSCO | 57.8% | 56.1% | 58.1% |
| Complete & duplicate copy  BUSCO | 30.4% | 31.7% | 30.40% |
| Fragmented BUSCO | 1% | 1.3% | 1.7% |
| Missing BUSCO | 10.80% | 10.90% | 9.8% |

With reference genome:

B73_v4 (with Mo17 as reference genome):

quast-lg.py --k-mer-stats --eukaryote --est-ref-size 2200000000 --debug --threads 36 --min-contig 0 --large B73_v4_scaffolds.fasta -r Zm-Mo17-Scaffolds-CAU-1.0.fa -o b73

Mo17 (with B73_v4 as reference genome):

quast-lg.py --k-mer-stats --eukaryote --est-ref-size 2200000000 --debug --threads 36 --min-contig 0 –large Zm-Mo17-Scaffolds-CAU-1.0.fa -r B73_v4_scaffolds.fasta -o mo17

W22 (with B73_v4 as reference genome):

quast-lg.py --k-mer-stats --eukaryote --est-ref-size 2200000000 --debug --threads 36 --min-contig 0 --large W22_NRgene_scaffolds.fasta -r B73_v4_scaffolds.fasta -o w22

**Table S2**. QUAST-LG output with reference genome as input

| **Metrics** | **B73_v4** | **Mo17** | **W22** |
| --- | --- | --- | --- |
| # misassemblies | 152330 | 157227 | 158307 |
| # misassembled contigs | 549 | 1602 | 271 |
| Misassembled contigs length | 2126603898 | 2159635497 | 2133381234 |
| # local misassemblies | 66250 | 69150 | 58599 |
| # scaffold gap ext. mis. | 48 | 32 | 535 |
| # scaffold gap loc. mis. | 378 | 354 | 22749 |
| # possible TEs | 9842 | 10156 | 11332 |
| # unaligned mis. contigs | 12 | 216 | 4 |
| # unaligned contigs | 1 + 552 part | 15 + 1894 part | 1 + 259 part |
| Unaligned length | 431622376 | 442931989 | 409379743 |
| Genome fraction (%) | 62.018 | 63.448 | 64.166 |
| Duplication ratio | 1.278 | 1.303 | 1.278 |
| # mismatches per 100 kbp | 2199.68 | 2256.19 | 1976.02 |
| # indels per 100 kbp | 94.38 | 104.28 | 71.92 |
| Largest alignment | 4340541 | 2999268 | 2877268 |
| Total aligned length | 1664946941 | 1696765448 | 1679950589 |
| NA50 | 13565 | 13172 | 15014 |
| NGA50 | 12732 | 13908 | 14997 |
| NA75 | 2333 | 2119 | 2537 |
| NGA75 | 1399 | 2951 | 2529 |
| LA50 | 15166 | 16588 | 12367 |
| LGA50 | 16999 | 14812 | 12383 |
| LA75 | 99136 | 106741 | 94305 |
| LGA75 | 118936 | 92393 | 94446 |
| K-mer-based compl. (%) | 42.31 | 42.47 | 45.40 |

With reads:

The raw reads were downsampled to 20X coverage using seqtk and then used within the QUAST-LG pipeline for quick analysis.

B73_v4:

seqtk sample -s100 SRR2960981_1.fastq 0.2 > sub1.fq

seqtk sample -s100 SRR2960981_2.fastq 0.2 > sub2.fq

quast-lg.py --k-mer-stats --eukaryote --est-ref-size 2200000000 --debug --threads 36 --min-contig 0 --large B73_v4_scaffolds.fasta --pe1 sub1.fq --pe2 sub2.fq -o b73

Mo17:

cat SRR5826129_1.fastq SRR5826129_1.fastq > forward.fastq

cat SRR5826130_2.fastq SRR5826130_2.fastq > reverse.fastq

seqtk sample -s100 forward.fastq 0.15 > sub1.fq

seqtk sample -s100 reverse.fastq 0.15 > sub2.fq

quast-lg.py --k-mer-stats --eukaryote --est-ref-size 2200000000 --debug --threads 36 --min-contig 0 --large Zm-Mo17-Scaffolds-CAU-1.0.fa --pe1 sub1.fq --pe2 sub2.fq -o mo17

**Table S3:** QUAST-LG output with reads as input

| **Metrics** | **B73_v4** | **Mo17** |
| --- | --- | --- |
| Mapped (%) | 97.15 | 94.88 |
| Properly paired (%) | 94.73 | 89.68 |
| Singletons (%) | 0.19 | 0.23 |
| Misjoint mates (%) | 1.57 | 1.67 |
| Avg.coveragedepth | 93 | 102 |
| Coverage >= 1x (%) | 97.95 | 97.53 |
| Coverage >= 5x (%) | 97.52 | 96.88 |
| Coverage >= 10x (%) | 97.14 | 96.42 |

**REAPR:**

The raw reads were downsampled to 20X coverage using seqtk and then used within the REAPR pipeline for quick analysis.

Note: We didn’t have raw read information for W22 and REAPR needs the read information as input to work.

B73_V4:

reapr smaltmap -n 36 B73_v4_scaffolds.fasta B73sub1.fq B73sub2.fq B73out.smaltmap.bam

reapr pipeline B73_v4_scaffolds.fasta B73out.smaltmap.bam B73outdir

Mo17:

reapr smaltmap -n 36 Zm-Mo17-Scaffolds-CAU-1.0.fa Mo17sub1.fq Mo17sub2.fq Mo17out.smaltmap.bam

reapr pipeline Zm-Mo17-Scaffolds-CAU-1.0.fa Mo17out.smaltmap.bam B73outdir

**Table S4:** REAPR output

| **Reference free Metrics** | **B73_v4** | **Mo17** |
| --- | --- | --- |
| Total length | 2134339606 | 2182615441 |
| Number of sequences | 596 | 2560 |
| Mean sequence length | 3581106.72 | 852584.16 |
| Length of longest sequence | 39317442 | 32176138 |
| N50, n | 10679169, n = 62 | 10204498, 69 |
| N60, n | 8544608, n = 85 | 7989645, 94 |
| N70, n | 7103792, n = 113 | 5983233, 125 |
| N80, n | 4964772, n = 148 | 4640909, 166 |
| N90, n | 3077177, n = 204 | 2306856, 231 |
| N100, n | 5568, n = 596 | 1007, 2560 |
| Number of gaps | 2192 | 6490 |

| **Read based Metrics** | **B73_v4** | **Mo17** |
| --- | --- | --- |
| Error free bases | 0.03% | 0.19% |
| FDC errors within a contig | 0 | 0 |
| FDC errorsover a gap | 0 | 0 |
| Low fragment coverage within a contig | 6408154 | 2905114 |
| Low fragment coverage over a gap | 1083 | 105 |
| Low score regions | 0 | 0 |
| Links | 4608 | 18032 |
| soft clip | 2352 | 4402 |
| collapsed reapeats | 28138 | 172884 |
| Low read coverage | 54500 | 68771 |
| Low perfect coverage | 0 | 0 |
| Wrong read orientation | 1777214 | 3250371 |

**GenomeQC:**

Commands used in the pipeline (all these scripts can be obtained through the project GitHub repository):

Assembly (using just the genome assembly as input):

B73_v4:

python assembly_stats.py B73_v4_scaffolds.fasta B73_assembly_output 2200

python run_BUSCO_fasta.py --in B73_v4_scaffolds.fasta --out b73_busco -m genome --lineage embryophyta_odb9 maize -c 4 --blast_single_core

gt suffixerator -db B73_modified_header_names.fasta -indexname B73_modified_header_names.fasta -tis -suf -lcp -des -ssp -sds -dna

ltrharvest -index B73_modified_header_names.fasta -minlenltr 100 -maxlenltr 7000 -mintsd 4 -maxtsd 6 -motif TGCA -motifmis 1 -similar 85 -vic 10 -seed 20 -seqids yes > B73_modified_header_names.fasta.harvest.scn

LTR_FINDER_parallel -seq B73_modified_header_names.fasta -threads 36 -harvest_out -size 1000000 -time 300

cat B73_modified_header_names.fasta.harvest.scn

B73_modified_header_names.fasta.finder.combine.scn > B73_modified_header_names.fasta.rawLTR.scn

LTR_retriever -genome B73_modified_header_names.fasta -inharvest B73_modified_header_names.fasta.rawLTR.scn -threads 36

Mo17:

python assembly_stats.py Zm-Mo17-Scaffolds-CAU-1.0.fa Mo17_assembly_output 2200

python run_BUSCO_fasta.py --in Zm-Mo17-Scaffolds-CAU-1.0.fa --out mo17_busco -m genome --lineage embryophyta_odb9 maize -c 4 --blast_single_core

gt suffixerator -db Mo17_modified_header_names.fasta -indexname Mo17_modified_header_names.fasta -tis -suf -lcp -des -ssp -sds -dna

ltrharvest -index Mo17_modified_header_names.fasta -minlenltr 100 -maxlenltr 7000 -mintsd 4 -maxtsd 6 -motif TGCA -motifmis 1 -similar 85 -vic 10 -seed 20 -seqids yes > Mo17_modified_header_names.fasta.harvest.scn

LTR_FINDER_parallel -seq Mo17_modified_header_names.fasta -threads 36 -harvest_out -size 1000000 -time 300

cat Mo17_modified_header_names.fasta.harvest.scn

Mo17_modified_header_names.fasta.finder.combine.scn > Mo17_modified_header_names.fasta.rawLTR.scn

LTR_retriever -genome Mo17_modified_header_names.fasta -inharvest Mo17_modified_header_names.fasta.rawLTR.scn -threads 36

W22:

python assembly_stats.py W22_NRgene_scaffolds.fasta W22_assembly_output 2200

python run_BUSCO_fasta.py --in W22_NRgene_scaffolds.fasta --out w22_busco -m genome --lineage embryophyta_odb9 maize -c 4 --blast_single_core

gt suffixerator -db W22_modified_header_names.fasta -indexname W22_modified_header_names.fasta -tis -suf -lcp -des -ssp -sds -dna

ltrharvest -index W22_modified_header_names.fasta -minlenltr 100 -maxlenltr 7000 -mintsd 4 -maxtsd 6 -motif TGCA -motifmis 1 -similar 85 -vic 10 -seed 20 -seqids yes > W22_modified_header_names.fasta.harvest.scn

LTR_FINDER_parallel -seq W22_modified_header_names.fasta -threads 36 -harvest_out -size 1000000 -time 300

cat W22_modified_header_names.fasta.harvest.scn

W22_modified_header_names.fasta.finder.combine.scn > W22_modified_header_names.fasta.rawLTR.scn

LTR_retriever -genome W22_modified_header_names.fasta -inharvest W22_modified_header_names.fasta.rawLTR.scn -threads 36

**Table S5**: GenomeQC assembly output

| **Reference free Metrics** | **B73_v4** | **Mo17** | W22 |
| --- | --- | --- | --- |
| Number of scaffolds | 596 | 2560 | 306 |
| Total size of scaffolds | 2134339606 | 2182615441 | 2133868603 |
| Total scaffold length as percentage of assumed genome size | 97.01543664 | 99.20979277 | 96.9940274 |
| useful amount of scaffold sequences (>=25K nt) | 2134248774 | 2166421525 | 2132523330 |
| % of estimated genome that is useful | 97.01130791 | 98.47370568 | 96.9328786 |
| Longest scaffold | 39317442 | 32176138 | 83688764 |
| Shortest scaffold | 5568 | 1007 | 711 |
| Number of scaffolds > 1K nt | 596 | 2560 | 305 |
| Number of scaffolds > 10K nt | 591 | 2216 | 291 |
| Number of scaffolds > 100K nt | 366 | 475 | 130 |
| Number of scaffolds > 1M nt | 296 | 304 | 97 |
| Number of scaffolds > 10M nt | 69 | 69 | 62 |
| N50 | 10679169 | 10204498 | 35520101 |
| L50 | 62 | 69 | 19 |
| NG50 | 10214929 | 9989738 | 33636442 |
| LG50 | 66 | 70 | 20 |
| %A | 26.17515251 | 26.16202361 | 26.1136293 |
| %C | 23.08858481 | 23.04310739 | 22.9215831 |
| %G | 23.10360491 | 23.03462569 | 22.9355355 |
| %T | 26.1942841 | 26.15118015 | 26.125969 |
| Total Number of Ns | 30699779 | 35119661 | 40613559 |
| %N | 1.438373674 | 1.609063161 | 1.90328303 |
| Complete & single copy BUSCO | 89.30% | 88.80% | 89.90% |
| Complete & duplicate copy BUSCO | 6.10% | 6.70% | 5.60% |
| Fragmented BUSCO | 1.40% | 1.50% | 1.50% |
| Missing BUSCO | 2.80% | 2.90% | 3% |
| LAI | 25.75 | 25.17 | 19.25 |

Vector contamination check:

Annotation (using the genome annotation file and transcripts file):

B73_V4:

python gff3_stats.py Zea_mays.AGPv4.36.gff3 B73_annotation_output

run_BUSCO_gff.py --in B73_v4_transcripts.fasta --out B73_busco_annotation -m transcriptome –lineage embryophyta_odb9 -c 4 --blast_single_core

Mo17:

python gff3_stats.py Mo17.gff3 Mo17_annotation_output

run_BUSCO_gff.py --in Mo17_transcripts.fasta --out Mo17_busco_annotation -m transcriptome –lineage embryophyta_odb9 -c 4 --blast_single_core

W22:

python gff3_stats.py W22.gff3 W22_annotation_output

run_BUSCO_gff.py --in W22_transcripts.fasta --out W22_busco_annotation -m transcriptome –lineage embryophyta_odb9 -c 4 --blast_single_core

**Table S6**: GenomeQC annotation output

| **Reference free Metrics** | **B73_v4** | **Mo17** | W22 |
| --- | --- | --- | --- |
| Number of gene models (bp) | 39498 | 38620 | 40691 |
| Minimum gene length (bp) | 111 | 21 | 87 |
| Maximum gene length (bp) | 128402 | 146217 | 154495 |
| Average gene length (bp) | 4173 | 4076 | 4330 |
| Number of exons | 1209198 | 272323 | 313830 |
| Average number of exons per gene model | 30 | 7 | 7 |
| Average exon length (bp) | 284 | 297 | 292 |
| Number of transcripts | 133786 | 46530 | 51717 |
| Average number of transcripts per gene model | 3 | 1 | 1 |
| Number of gene models less than 200bp length | 37 | 1225 | 64 |
| Complete & single copy BUSCO | 47.10% | 69.7% | 65.1% |
| Complete & duplicate copy BUSCO | 49.60% | 23% | 25.1% |
| Fragmented BUSCO | 1.90% | 4.6% | 6% |
| Missing BUSCO | 1.4% | 2.7% | 3.8% |
